# Pallister–Hall syndrome diagnosed in a young man after an acute adrenal crisis

**DOI:** 10.1002/ccr3.6249

**Published:** 2022-08-22

**Authors:** Anis Grassa, Meriem Yazidi, Jihene Marrakchi, Chaima Bel Hadj Sliman, Ibtissem Oueslati, Melika Chihaoui

**Affiliations:** ^1^ Endocrinology Department, Faculty of Medicine of Tunis, La Rabta Hospital University of Tunis el Manar Tunis Tunisia; ^2^ Otolaryngology Department, Faculty of Medicine of Tunis, La Rabta Hospital University of Tunis el Manar Tunis Tunisia

**Keywords:** adrenal insufficiency, hamartoma, hypopituitarism, Pallister–Hall syndrome

## Abstract

Pallister–Hall syndrome (PHS) is a very rare genetic disorder. The diagnosis is usually suspected at the young age when a hypothalamic hamartoma is associated with polydactyly. Endocrine manifestations are mostly related to hypothalamic hamartoma and rarely reveal the disease. We report the case of an 18‐year‐old young man in whom the diagnosis of PHS was delayed until his hospitalization in the endocrinology department for acute adrenal insufficiency.

## Graphical abstract

Pallister‐Hall syndrome may be misdiagnosed given its extremely low prevalence. This syndrome can be revealed by an acute adrenal crisis.

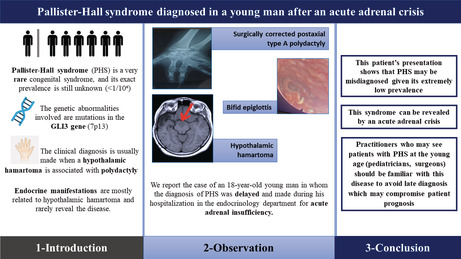

## 1 INTRODUCTION

Pallister–Hall syndrome (PHS) is a very rare congenital syndrome, and its exact prevalence is still unknown (<1/10^6^). It was first described in 1981 by Philip Pallister and Judith Hall. To date, only 100 patients have been described worldwide.^1^ The genetic abnormalities involved are mutations in the GLI3 gene (7p13) encoding a transcription factor activated by the Sonic hedgehog signaling pathway.^2^, ^3^, ^4^ The clinical diagnosis is usually made when a hypothalamic hamartoma is associated with polydactyly.^5^, ^6^ Additional symptoms and findings may include oro‐facial malformations and other abnormalities like genitourinary malformations.^7^, ^8^ Endocrine manifestations are mostly related to hypothalamic hamartoma. They consist of hypopituitarism, which can affect one or more pituitary axes, and precocious puberty.^8^ Here, we report the case of an 18‐year‐old young man in whom the diagnosis of PHS was delayed until his hospitalization in the endocrinology department for acute adrenal insufficiency.

## 2 CASE PRESENTATION

The patient was an 18‐year‐old young man who presented to the emergency room with clinical and biological features of an adrenal crisis (abdominal pain, vomiting, hypoglycemia, hypotension, and hyponatremia). The clinical course was good after intravenous hydrocortisone replacement and infusion with physiological saline solution. The medical history revealed several surgeries: a corrective one for meso‐axial polydactyly, a surgery for testicular ectopia and for a hypothalamic tumor diagnosed at the age of 3 years complicated with hypopituitarism. He received growth hormone for 2 years from the age of 12. He was on hormone replacement therapy for hypothyroidism and adrenal insufficiency, and he stopped his treatment for a week. He had no medical follow‐up for several years. The patient's family history was unknown as he was an adopted child.

**FIGURE 1:**
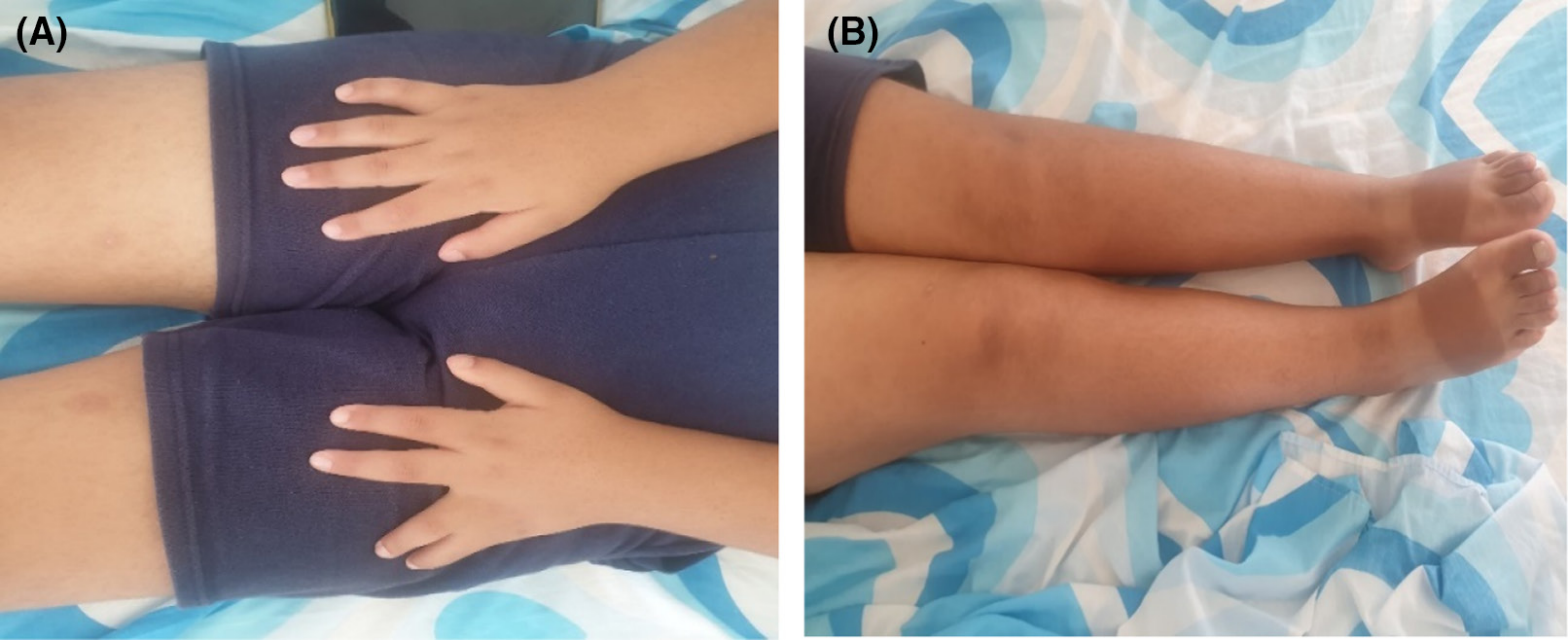
(A) Surgically corrected postaxial polydactyly; (B) Lower limb inequality

**FIGURE 2:**
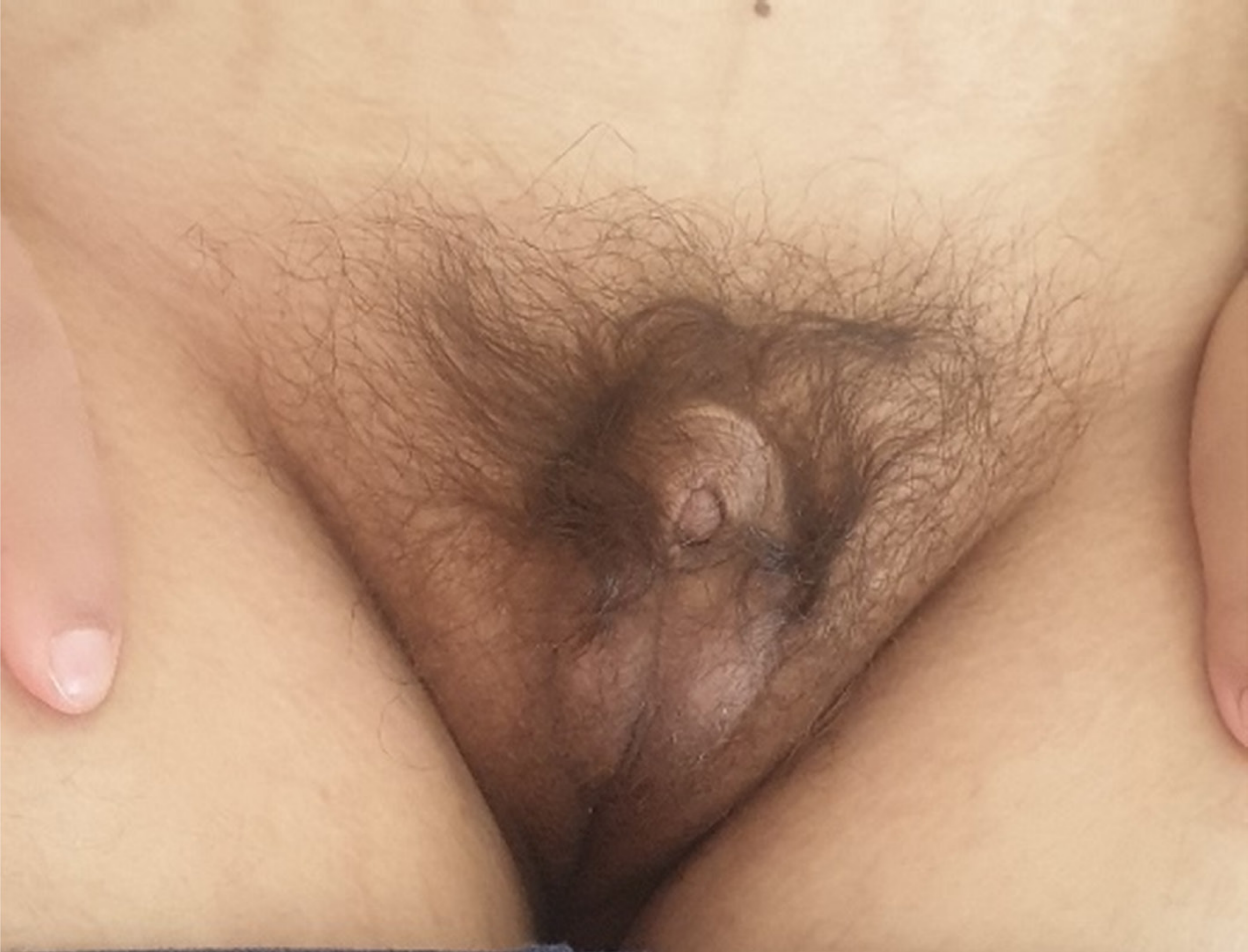
Micropenis and hypoplastic testes

**FIGURE 3:**
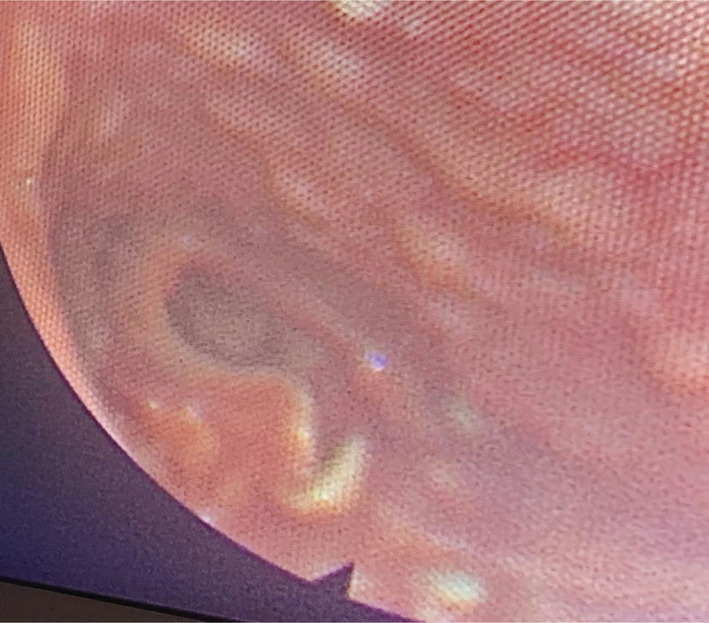
Bifid epiglottis

**FIGURE 4:**
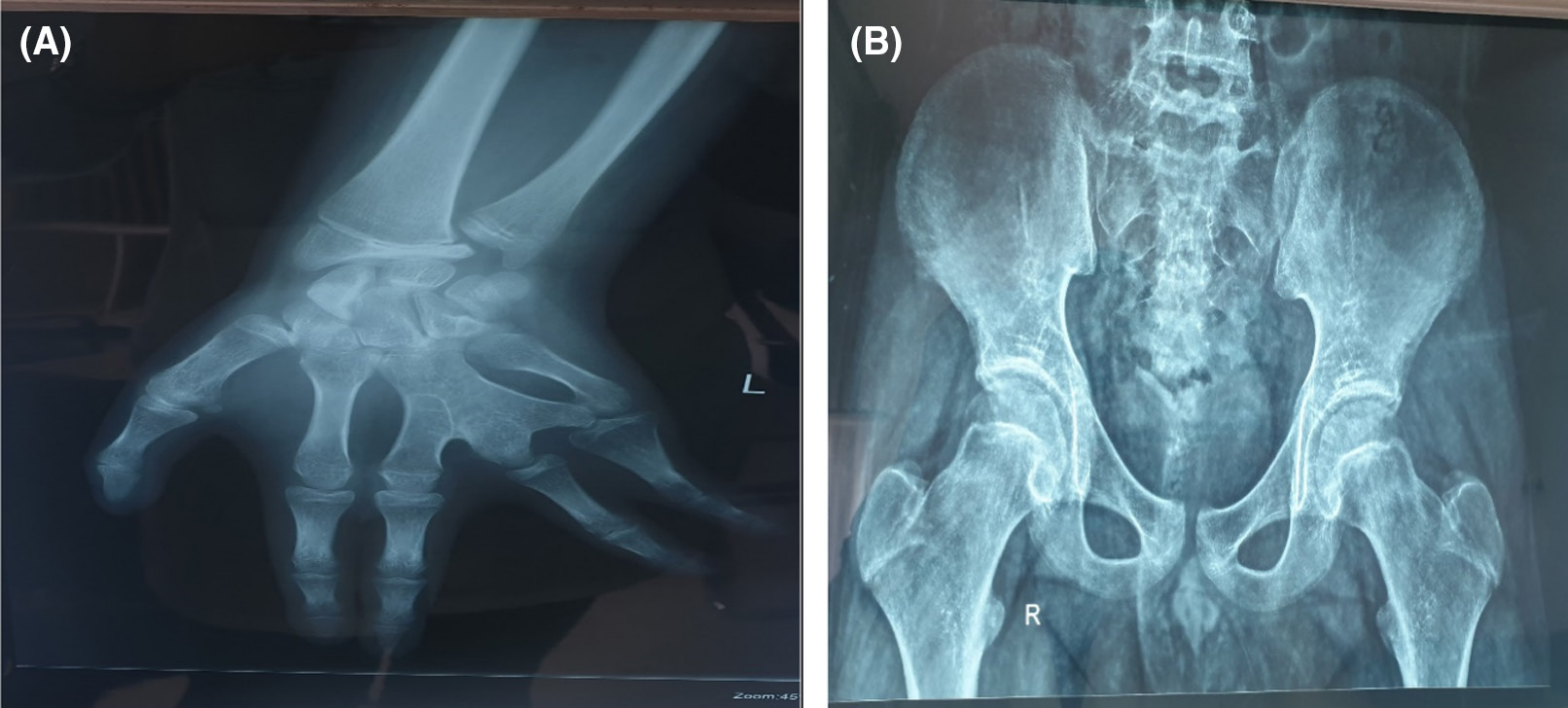
(A) Surgically corrected postaxial type A polydactyly; (B) Risser stage 4 and bone demineralization

**FIGURE 5:**
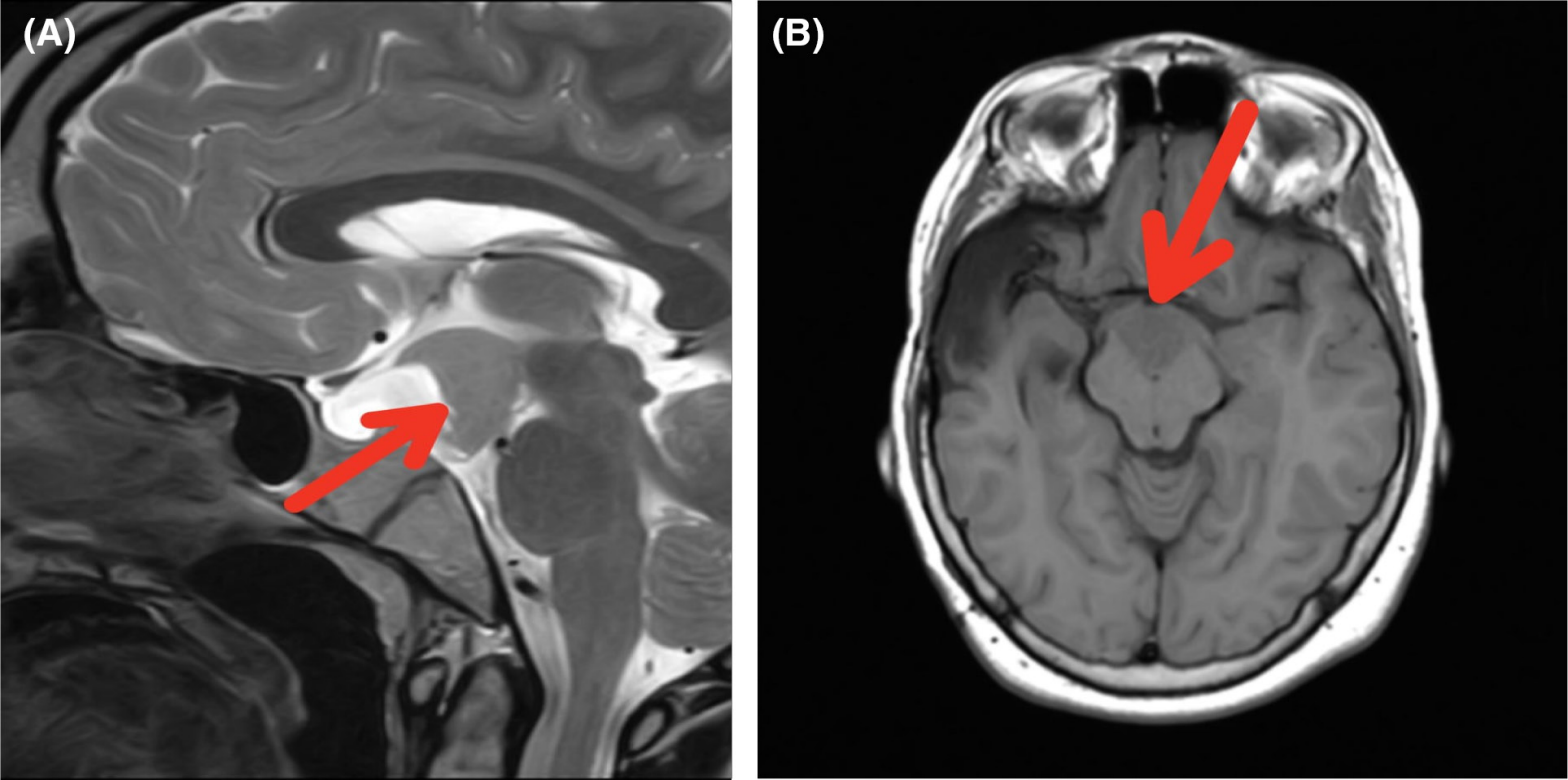
(A) Brain MRI showing a sagittal view of hypothalamic hamartoma; (B) Brain MRI showing an axial view of hypothalamic hamartoma

Physical examination showed a height of 157 cm (−3 SD), a weight of 68 kg, and a BMI of 27.5 kg/m^2^. We noted a surgically corrected postaxial polydactyly and an inequality of the two lower limbs (Figure 1). Examination of the external genitalia revealed a micropenis and hypoplastic testes (Figure 2). PHS was suspected and further investigations were then performed. An otolaryngology examination showed a bifid epiglottis as well as a laryngeal cleft (Figure 3). X‐rays of the left hand revealed a surgically corrected postaxial type A polydactyly (Figure 4A). Pelvic X‐ray showed bone demineralization and the Risser stage was 4 (Figure 4B). Ultrasounds of the heart, the abdomen, and kidneys were normal. The pelvic ultrasound showed hypoplastic testes. Brain MRI revealed a sellar and suprasellar mass measuring 28 × 25 × 24 mm corresponding to a hypothalamic hamartoma (Figure 5). On biochemical evaluation, the renal and hepatic functions, blood count, serum calcium, and serum phosphorus results were normal. The pituitary assessment showed a thyrotropin and gonadotropin deficiencies (Table 1).

**TABLE 1:** Pituitary function results

Parameter	Data	Normal reference range
FT4 (pmol/L)	7.1	9.1–19.5
TSH (mIU/L)	0.04	0.1–4.95
Testosterone (nmol/L)	<0.45	10–33.3
FSH (IU/L)	0.49	3–8
LH (IU/L)	0.13	0.6–12
PRL (pmol/L)	81.3	<1086

Abbreviations: FSH, follicle stimulating hormone; FT4, Free Thyroxine; LH, luteinizing hormone; PRL, prolactin; TSH, thyroid‐stimulating hormone.

## 3 DISCUSSION

The diagnosis of PHS is usually suspected at a young age based on the association of polydactyly and hypothalamic hamartoma.^1^, ^5^, ^6^, ^7^, ^8^, ^9^ However, due to its extreme rarity, the diagnosis may be unrecognized for a long time. Endocrine manifestations can then be in the foreground and reveal the diagnosis as was the case for our patient who presented with an adrenal crisis. Adrenal insufficiency is, in most cases, linked to a hypothalamic hamartoma which is noted in 90%–100% of cases of PHS.^4^, ^9^ Cases of congenital adrenal hypoplasia have also been reported.^8^, ^9^ Hall's review reports two PHS cases of death at birth secondary to adrenal insufficiency.^10^ Hypothalamic hamartoma leads to one or more pituitary axes deficiencies. However, corticotropin deficiency seems rare in this syndrome, especially in familial forms.^8^ Growth hormone deficiency appears to be the most common pituitary deficiency, but short stature in PHS may also be the result of intrauterine growth retardation.^9^ Panhypopituitarism has only been reported in one case.^9^ As shown in Table 1, the hormonal deficits presented by our patient are of central origin. However, we cannot conclude whether they are related to the hamartoma or to hypothalamic–pituitary surgery.

It should also be noted that the characteristics of hamartoma in PHS differ from those of non‐syndromic hamartomas. Epilepsy, behavioral disturbances, and gelastic seizures are less common in PHS and respond better to treatment if they are present.^8^, ^11^, ^12^ Our patient had no history of seizures and had no behavioral disturbances. Precocious puberty can also reveal hamartoma. Its prevalence seems to be less important in hamartomas associated with PHS than in non‐syndromic hamartomas.^11^ Asymptomatic hamartoma detected incidentally on imaging have also been reported.^13^ Thus, hamartomas in PHS usually only require monitoring and surgery is usually not indicated.

The diagnosis of PHS requires screening for associated abnormalities. A bifid epiglottis was found on the otolaryngology examination of our patient. This malformation is present in 40%–100% of cases in PHS and is most often asymptomatic.^8^, ^9^, ^13^, ^14^, ^15^ However, some patients present with more severe posterior laryngeal clefts, leading to potentially fatal respiratory failure.^1^, ^8^ Bifid epiglottis is a rarely isolated birth defect, and its detection should suggest the diagnosis of PHS.^14^ Heart, lung, and kidney malformations have also been reported in PHS. Cardiac, abdominal, and renal ultrasound were normal in our patient. Urogenital abnormalities are reported in almost half of cases such as micropenis, hypospadias, and uterovaginal aplasia.^4^, ^9^ The micropenis in the present case can be related to the syndrome but can also be explained by congenital hypogonadotropic hypogonadism.

The management in the present case will be limited to hormonal replacement therapy. The delay in the diagnosis and management of growth hormone deficiency has compromised the final height of the patient. An early treatment with growth hormone may have given him normal growth. Galasso et al.^16^ described a case of a PHS boy treated from the age of 3 years and for 7 years with growth hormone. His final height was 5.5 cm above his target height.

## 4 CONCLUSION

This patient's presentation shows that PHS may be misdiagnosed given its extremely low prevalence. This syndrome can be revealed by an acute adrenal crisis. Practitioners who may see patients with PHS at the young age (pediatricians, surgeons) should be familiar with this disease to avoid late diagnosis which may compromise patient prognosis.

## AUTHOR CONTRIBUTIONS

Anis Grassa have made substantial contributions to conception and design, acquisition of data, and analysis and interpretation of data. Meriem Yazidi performed acquisition of data and manuscript drafting. Melika Chihaoui critically revised the article for important intellectual content. All authors were involved in the management of this patient and the revision of the manuscript and approved the final version.

## CONFLICT OF INTEREST

There are no conflicts of interest.

## CONSENT

Written informed consent was obtained from the patient to publish this report in accordance with the journal's patient consent policy.

## Data Availability

Data available on request due to privacy/ethical restriction: The data that support the findings of this study are available on request from the corresponding author. The data are not publicly available due to privacy or ethical restrictions.
